# Integrated Experimental and Preliminary In Silico Study of Myrtenyl Dihydrocaffeate: Biocatalytic Synthesis Optimization, Antioxidant Evaluation, and Oxidative Stabilization of Rapeseed Oil

**DOI:** 10.3390/biom16071034

**Published:** 2026-07-15

**Authors:** Bartłomiej Zieniuk, Jakub Gielmuda, Chimaobi James Ononamadu

**Affiliations:** 1 Department of Chemistry, Institute of Food Sciences, Warsaw University of Life Sciences-SGGW, 159C Nowoursynowska Str., 02-776 Warsaw, Poland; 2 Faculty of Biology and Biotechnology, Warsaw University of Life Sciences-SGGW, 159C Nowoursynowska Str., 02-776 Warsaw, Poland; 3 Department of Biochemistry and Forensic Science, Nigeria Police Academy Wudil, Kano P.O. Box 14830, Nigeria; ononamaducj@polac.edu.ng

**Keywords:** myrtenyl dihydrocaffeate, enzymatic esterification, *Candida antarctica* lipase B, antioxidant activity, molecular docking

## Abstract

Dihydrocaffeic acid (DHCA) is a naturally occurring phenolic acid with recognized antioxidant and biological properties. However, its relatively high polarity limits its applicability in lipid-based systems. In this study, myrtenyl dihydrocaffeate was synthesized through lipase-catalyzed esterification of DHCA with myrtenol using immobilized *Candida antarctica* lipase B. The reaction conditions were optimized using response surface methodology based on a central composite design, yielding an experimental ester yield of 39.94 ± 1.16%. The synthesized ester was characterized by NMR spectroscopy and subsequently evaluated using a combination of experimental and in silico approaches. Antioxidant activity was determined by DPPH^•^ and ABTS^•+^ radical scavenging assays, while oxidative stabilization of rapeseed oil was assessed by pressure differential scanning calorimetry (PDSC). Antimicrobial activity was evaluated using disk diffusion, minimum inhibitory concentration (MIC), and minimum bactericidal concentration (MBC) assays. In silico studies included ADMET profiling, PASS bioactivity prediction, protein target prediction, and molecular docking. These computational analyses were used only as hypothesis-generating tools because the predicted protein targets had low target-probability scores and were not experimentally validated. Myrtenyl dihydrocaffeate retained substantial antioxidant activity and significantly improved the oxidative stability of rapeseed oil, exhibiting protection factors comparable to those of DHCA. The ester also demonstrated mild antimicrobial activity against selected Gram-positive bacteria. Overall, the results indicate that lipophilization of DHCA with myrtenol is an effective strategy for developing lipophilic antioxidant derivatives for lipid-based food, cosmetic, or topical formulations, while the predicted molecular targets require experimental validation.

## 1. Introduction

Oxidation-driven damage remains a major challenge in both biology and industry. In living organisms, reactive oxygen species (ROS) are produced regularly during metabolism and signaling. However, excess ROS can overwhelm cellular defenses, causing oxidative stress that damages lipids, proteins, and nucleic acids [1]. Factors like tobacco smoke, air pollution, and certain foods can increase ROS levels and oxidative stress [2]. This stress has been associated with aging and various chronic diseases [2,3,4,5]. Additionally, interest in antioxidants is growing due to their protective effects and the benefits of higher dietary intake [6,7].

In industrial environments, oxidation adversely affects many products, particularly foods, cosmetics, and pharmaceuticals. Lipid-rich matrices are especially vulnerable: rancidity results from complex processes, including radical oxidation triggered by light, oxygen, heat, metal ions, or enzymes. These processes initiate chain reactions that generate lipid radicals and secondary volatile compounds, such as aldehydes, ketones, and fatty acids [8]. Such changes deteriorate sensory qualities and functional properties and may pose health risks when oxidized fats are ingested, although evidence remains limited [9]. To improve lipid stability, strategies include UV-protective packaging and the addition of external antioxidants, with growing interest in natural, consumer-preferred additives [10]. Although synthetic stabilizers such as butylated hydroxytoluene (BHT) are effective, concerns about toxicity and consumer acceptance are driving research into natural alternatives [10,11].

Phenolic acids and polyphenols are important natural antioxidants that effectively neutralize free radicals, offering protective benefits [6]. Dihydrocaffeic acid (DHCA), a hydroxyphenylpropanoic acid and the saturated analog of caffeic acid, appears in various plants’ phenolic profiles but is less common than caffeic acid [12]. DHCA exhibits diverse bioactivities, including antimicrobial, cytoprotective, anti-inflammatory, and metal-chelating properties [12,13]. Importantly for antioxidant use, DHCA exhibits radical-scavenging activity, primarily via hydrogen donation from its catechol hydroxyl groups [14,15].

Terpenoids are prevalent plant metabolites widely used in industry, with terpene alcohols serving as key components of oils used in cosmetics, flavorings, and traditional remedies [16,17]. Many terpene alcohols are generally considered safe, display antimicrobial and antioxidant activities, and, thanks to their hydrophobicity, are ideal for lipid-based formulations. Their hydroxyl groups can also facilitate esterification with phenolic acids, producing more lipophilic derivatives. Myrtenol, a bicyclic monoterpene alcohol found in essential oils (e.g., *Myrtus*, *Artemisia*, and *Rhodiola* genus), is known for its antioxidant and antibacterial properties and is used in cosmetics [18].

An environmentally friendly approach for creating these conjugates is enzymatic esterification, which is increasingly considered a component of “green synthesis” because it demands less energy and milder conditions than many conventional chemical processes [19]. Nonetheless, practical challenges remain in selecting reaction media that dissolve both the relatively polar phenolic acid and the hydrophobic terpene alcohol without hindering enzyme activity, thereby requiring systematic optimization.

Lipase-catalyzed esterification is widely used to synthesize lipophilic derivatives of phenolic acids, including esters of caffeic, ferulic, dihydrocaffeic, and related hydroxycinnamic or hydroxyphenylpropanoic acids [20,21]. This strategy is attractive because it can improve the compatibility of phenolic antioxidants with lipid-based systems while preserving the phenolic moiety responsible for radical-scavenging activity [21,22]. Recent studies on lipophilic derivatives of phenolic acids have also shown that increasing lipophilicity can enhance their utility as antioxidant additives in edible oil systems, including thermoxidative stabilization of sunflower oil [22]. However, these reactions are often more challenging than esterification of simple aliphatic acids. Phenolic acids contain polar carboxyl and hydroxyl groups, have limited solubility in many hydrophobic media, and may interact unfavorably with the enzyme microenvironment [21]. Moreover, lipases such as CALB generally display high efficiency toward many aliphatic substrates, but their specificity toward bulky or aromatic substrates may be lower and more dependent on reaction conditions. Therefore, parameters such as solvent type, substrate molar ratio, water activity, enzyme loading, temperature, and reaction time are critical for achieving satisfactory yields and often require systematic optimization [20,21]. In this context, response surface methodology is particularly useful because it allows simultaneous evaluation of several reaction parameters and their interactions rather than optimizing each factor independently [20].

While DHCA derivatization and terpene alcohols are individually suitable for developing lipophilic antioxidants, a comprehensive approach that (i) optimizes CALB-mediated synthesis, (ii) directly measures bioactivity, and (iii) explores structure–activity relationships through docking-based, target-specific hypotheses is still limited for DHCA–terpene esters. As a result, many reports on the activity differences in newly created lipophilic DHCA derivatives are largely descriptive, lacking clear molecular insights and guidance for rational design. This research combines experimental and computational methods to study myrtenyl dihydrocaffeate, systematically refine CALB-catalyzed esterification, assess antioxidant and antimicrobial properties, evaluate oxidative stabilization in rapeseed oil, and conduct preliminary in silico analyses to generate hypotheses about potential structure–activity relationships.

## 2. Materials and Methods

### 2.1. Materials

*Candida antarctica* lipase B (product no. L4777), along with other solvents and chemicals, was obtained from Merck Life Science Sp. z o.o. (Poznań, Poland). Rapeseed oil (Bunge Polska Sp. z o.o., Kruszwica, Poland) was bought from a supermarket in Warsaw, Poland.

### 2.2. Enzymatic Esterification of Dihydrocaffeic Acid

**Figure 1:**
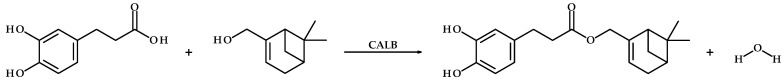
Scheme of enzymatic esterification of dihydrocaffeic acid with (1*R*)-(−)-myrtenol, catalyzed by immobilized lipase B from *C. antarctica* (CALB).

Esterification of dihydrocaffeic acid (DHCA) with myrtenol was performed using immobilized *C. antarctica* lipase B (CALB) as the biocatalyst (Figure 1). Reactions were carried out in 50 mL Erlenmeyer flasks using methyl *tert*-butyl ether (MTBE, 20 mL) as the reaction medium. MTBE was selected as the fixed reaction medium based on preliminary solubility considerations and previous observations that CALB activity is strongly affected by the organic solvent used, with substrate solubility an important criterion for solvent selection [23]. DHCA was used at a fixed amount (0.0020 mol), and the amount of myrtenol was adjusted according to the planned molar ratio (alcohol:DHCA). Enzyme concentration was expressed as % (*w*/*w*) relative to the mass of DHCA and was set according to the experimental design. Reaction mixtures were incubated in a laboratory orbital shaker at 200 rpm under the specified temperature and reaction time.

To quantify and optimize the effects of key variables on ester formation, a four-factor central composite design (CCD) was applied. The design comprised 18 experiments (8 factorial points, 8 axial points, and 2 center-point replicates). The investigated factors were: A—temperature (°C), B—enzyme concentration (% *w*/*w* relative to DHCA mass), C—reaction time (h), and D—molar ratio (alcohol:DHCA, mol/mol). Axial points were defined using α = 1.68, giving the factor ranges summarized in Table 1. The response was expressed as isolated ester yield (%), calculated from the mass of purified product relative to the theoretical yield of ester from the limiting reagent. Because the esterification proceeds with 1:1 stoichiometry between DHCA and myrtenol, DHCA was the limiting reagent in reactions performed at myrtenol:DHCA ratios ≥ 1:1, whereas myrtenol was the limiting reagent in Experiment 15, performed at a myrtenol:DHCA ratio of 0.32:1.

After the reaction, the immobilized enzyme was removed by filtration. MTBE was evaporated under reduced pressure using a rotary evaporator (Büchi Rotavapor R-200, Büchi, Flawil, Switzerland). The crude product was purified by silica gel column chromatography on a glass column measuring 400 mm in length, 20 mm in internal diameter, and 125 mL in capacity. Approximately 35 g of silica gel 60 (0.040–0.063 mm; 230–400 mesh) served as the stationary phase. Chloroform:methanol (9:1, *v*/*v*) served as the mobile phase. Fractions containing the target ester were identified by thin-layer chromatography (TLC) on aluminum TLC plates coated with silica gel 60 and a fluorescent indicator, visualized under UV light at 254 nm. Relevant fractions were combined, dried over anhydrous MgSO_4_, filtered, and the solvent was removed under reduced pressure. The ester was recrystallized from n-heptane. The crystals were dried at 40 °C for 16 h and weighed to determine the isolated yield. Product identity was confirmed by ^1^H and ^13^C NMR spectroscopy (Bruker AVANCE 500 MHz, Billerica, MA, USA) using CDCl_3_ as the solvent and TMS as the internal standard. Chemical shifts (δ) are reported in ppm.

^1^H NMR (500 MHz, CDCl_3_) δ 6.77–6.69 (m, 3H, Ar–H), 5.53–5.52 (m, 1H, =CH–), 4.46–4.44 (m, 2H, OCH_2_), 2.83 (m, 2H, ArCH_2_–), 2.62–2.59 (m, 2H, –CH_2_–C(O)O–), 2.40–1.90 (m, aliphatic H), 1.27 (s, 3H, CH_3_), 1.16–1.14 (m, 1H), 0.80 (s, 3H, CH_3_).

^13^C NMR (126 MHz, CDCl_3_) δ 173.66, 143.60, 142.71, 142.06, 133.29, 121.80, 120.56, 115.39, 115.36, 67.40, 43.55, 40.66, 38.02, 36.21, 31.46, 31.26, 30.31, 26.11, 21.04.

Optical rotation was not determined, therefore, the stereochemical assignment of the myrtenyl fragment was based on the use of commercially available (1*R*)-(-)-myrtenol as the starting alcohol and was not independently verified. Since esterification occurs through the hydroxyl group of myrtenol, no change in the configuration of the myrtenyl fragment was expected under the applied reaction conditions.

### 2.3. Absorption, Distribution, Metabolism, Excretion, and Toxicity (ADMET) Properties Prediction

The ADMET properties of the newly synthesized esters and the parent molecules were evaluated using SwissADME [24,25] and pkCSM [26], using the canonical SMILES representations of the compounds. The following parameters were assessed: Absorption—water solubility, Caco-2 permeability, human intestinal absorption (%), and P-glycoprotein substrate/inhibitor status; Distribution—volume of distribution (VDss), blood–brain barrier permeability (logBB), and CNS permeability (logPS); Metabolism—CYP450 isoforms (CYP3A4, CYP2D6, and CYP2C9) substrate/inhibition potential; Excretion—total clearance (log mL/min/kg) and renal OCT2 substrate status; Toxicity—AMES toxicity, maximum tolerated dose (human), hERG I inhibitor, hERG II inhibitor, oral rat acute toxicity (LD_50_), oral rat chronic toxicity (LOAEL), hepatotoxicity, skin sensitization, *T. pyriformis* toxicity, and minnow toxicity; and Drug-likeness—Lipinski, Veber, Ghose, Egan and Muegge rules, as well as Abbot bioavailability score.

### 2.4. Bioactivity Prediction

To explore the potential bioactivity of the newly synthesized ester, bioactivity prediction was conducted using the PASS application (Prediction of Activity Spectra for Substances) [27]. This is a web-based application that predicts a spectrum of biological activities based on structure–activity relationships [28]. The canonical SMILES representation of the ester was submitted to the PASS online platform via the SMILES query box. A predicted activity was considered significant where the probability of activity (Pa) exceeded the probability of inactivity (Pi). A threshold of Pa ≥ 0.6 was applied to ensure high-confidence predictions. These predicted activities guided the identification of biologically relevant targets and informed subsequent molecular docking studies.

### 2.5. Protein Target Prediction

The prediction of potential targets for the newly synthesized ester was performed using SwissTargetPrediction [29] and SEA (Similarity Ensemble Approach) [30], two web-based applications that use chemical similarity principles to identify likely protein targets. The SMILES representations of the compounds were submitted, and the predicted targets were retrieved along with their associated probability scores. Targets were prioritized based on their relevance to the predicted biological activities from PASS analysis and their consistency with experimentally validated targets of the parent compounds reported in the literature. Because the predicted target probabilities were low, the selected targets were treated only as exploratory, hypothesis-generating candidates. They were not considered experimentally confirmed molecular targets of myrtenyl dihydrocaffeate.

### 2.6. Molecular Docking

For the exploratory molecular docking studies, proteins associated with inflammation-related pathways were selected as hypothetical targets, namely p38 MAP kinase (MAPK14), human 12-lipoxygenase (ALOX12), and transient receptor potential cation channel subfamily V member 1 (TRPV1). The docking analysis was not intended to confirm target engagement but to provide preliminary structural hypotheses for future experimental studies. The X-ray crystallographic structures of MAPK14 (1OVE), ALOX12 (8HGZ), and TRPV1 (8GFA), together with their co-crystallized ligands serving as controls, were retrieved in PDB format from the RCSB Protein Data Bank [31]. Protein preparation was carried out in the Molecular Operating Environment (MOE) using the QuickPrep function. The preparatory process involved removal of water molecules and other co-crystallized molecules, protonation, assignment of partial charges, and energy minimization. The fully optimized three-dimensional protein structures were subsequently saved in MOE format for docking analysis.

The docking sites for all target proteins were defined based on the binding regions occupied by their respective co-crystallized ligands, except for Human 12-lipoxygenase (ALOX12) (8HGZ), where the binding pocket was predicted using the Site Finder function in MOE.

The validation of the docking protocol was performed by re-docking of the isolated co-crystallized ligands into the active sites of their corresponding target proteins. The procedure was repeated using different scoring functions, including ASE, Affinity dG, Alpha HB, Electron Density, GBVI/WSA dG, and London dG. The resulting docking poses were compared with the experimentally determined binding conformations from X-ray crystal structures. A root mean square deviation (RMSD) value ≤ 2.0 Å relative to the native binding pose of the control ligand was considered acceptable for validation of the docking protocol [32]. Since the scoring functions consistently generated docking poses within the acceptable RMSD threshold, the default London dG/GBVI/WSA dG scoring function with induced fit refinement was adopted for the study.

Docking simulations were performed in MOE according to a previously described method with slight modifications [33]. The ligands were docked into the target proteins using the triangular matcher rigid receptor approach, which is the default docking algorithm in MOE, while ligand ranking was performed using the London dG and GBVI/WSA dG scoring functions on Intel Core i7 processors operating at 2.00 GHz and 2.60 GHz. The resulting ligand-binding poses, along with their respective docking scores, were saved in the MDB database format. The visualizations of the protein–ligand interactions were performed using Discovery Studio version 16.0 (Dassault Systèmes, Paris, France). Additionally, the docking scores of the best-ranked ligand conformations were normalized using size-independent ligand efficiency (SILE) values [33].

### 2.7. ABTS^•+^ Radical Cation Scavenging Assay

ABTS^•+^ scavenging activity was measured following the method described by Moreira [34]. A 7 mM ABTS solution was prepared in distilled water and mixed with ammonium persulfate to achieve a final persulfate concentration of 2.45 mM. The mixture was incubated for 16 h in the dark at room temperature to generate ABTS^•+^ radicals. The resulting ABTS^•+^ solution was then diluted with distilled water to reach an absorbance of 0.700 ± 0.020 at 734 nm. Stock solutions of DHCA, myrtenyl dihydrocaffeate, myrtenol, and BHT (1 mM) were prepared in ethanol. For each assay, 40 µL of the tested compound solution was added to 4.0 mL of ABTS^•+^ solution. After 6 min of incubation, absorbance was measured at 734 nm using distilled water as a reference. Radical scavenging activity was evaluated relative to a control sample, and IC_50_ values were calculated from concentration–response curves.

### 2.8. DPPH^•^ Radical Scavenging Assay

Antioxidant activity was assessed using the DPPH^•^ free radical scavenging assay, modified from the method described by Zieniuk et al. [35]. A 0.004% (*w*/*v*) DPPH^•^ solution was prepared with methanol, along with 1 mM stock solutions of DHCA, myrtenyl dihydrocaffeate, myrtenol, and BHT. The compound solution tested was mixed with the DPPH^•^ solution at a 1:9 (*v*/*v*) ratio. Following 30 min of incubation in the dark, absorbance was measured at 517 nm using a UV–Vis spectrophotometer (BRAIC, Beijing, China). IC_50_ values were calculated as the concentration needed to decrease the initial DPPH^•^ signal by 50%.

### 2.9. Fatty Acid Composition Analysis by GC–FID

The fatty acid profile of rapeseed oil was analyzed using gas chromatography with flame ionization detection (GC–FID) on a YL6100 GC system with Clarity software, version 7 (Young Lin, Anyang, Korea), equipped with an FID detector and a BPX-70 capillary column. Fatty acid methyl esters (FAME) were prepared following EN ISO 5509:2001 [36]. Nitrogen served as the carrier gas at a steady flow rate of 1.0 mL/min (constant flow mode). Samples were injected in split mode (split ratio 1:50). The oven temperature program was: 70 °C for 0.5 min; ramped at 15 °C/min to 160 °C; then increased at 1.1 °C/min to 200 °C and held for 12 min; finally, ramped at 30 °C/min to 225 °C and held for 1 min. Injector and detector temperatures were set at 225 °C and 250 °C, respectively. Each fatty acid was identified by comparing its retention time to a certified reference standard mixture (Supelco 37 Component FAME Mix, Sigma-Aldrich, Bellefonte, PA, USA). Results are presented as the percentage of each fatty acid relative to the total fatty acids.

### 2.10. Oxidative Stability Analysis by Pressure Differential Scanning Calorimetry (PDSC)

To evaluate oxidative stability, rapeseed oil samples were prepared as follows: (a) control oil without additives, (b) oil supplemented with DHCA, (c) oil supplemented with myrtenyl dihydrocaffeate, and (d) oil supplemented with myrtenol. In each case, the amount of additive was 0.01 mmol to 30 mL of oil. Oxidative stability was determined using pressure differential scanning calorimetry (PDSC) with a DSC Q20P calorimeter (TA Instruments, New Castle, DE, USA). An empty open aluminum pan was used as the reference. Approximately 3–4 mg of each oil sample was weighed into an open aluminum pan and subjected to isothermal oxidation under an oxygen pressure of 1350–1400 kPa. Measurements were performed at four temperatures: 110, 120, 130, and 140 °C. For each temperature, the time required to reach the maximum heat flow rate (τ_max_, min) was recorded and used as an indicator of oxidative stability. All measurements were carried out in triplicate, and the results are presented as mean values ± standard deviation.

The protection factor (PF) was calculated according to the following Equation (1):

(1) $$PF=\frac{\tau_{max(sample)}}{\tau_{max(control)}}$$

where τ_max(sample)_ and τ_max(control)_ represent the maximum oxidation times of the supplemented and control oils, respectively, determined at the same temperature.

The oxidation reaction rate coefficient (k, min^−1^) was calculated as the reciprocal of the maximum oxidation time:

(2) $$k=\frac{1}{\tau_{max}}$$

The temperature dependence of the oxidation process was described using the Arrhenius equation:

(3) $$k=Zexp\left(-\frac{E_{a}}{RT}\right)$$

where k is the reaction rate coefficient (min^−1^), Z is the pre-exponential factor (min^−1^), E_a_ is the activation energy (kJ mol^−1^), R is the universal gas constant (8.314 J mol^−1^ K^−1^), and T is the absolute temperature (K).

The Arrhenius parameters were determined by linear regression of the logarithmic form of the Arrhenius equation:

(4) $$ln\;k=ln\;Z-\frac{E_{a}}{R}\frac{1}{T}$$

The activation energy (E_a_), pre-exponential factor (Z), and coefficient of determination (R^2^) were calculated from the slope and intercept of the regression line obtained by plotting ln k against 1/T. Linear regression analysis for Arrhenius plots was performed using Excel 365 (Microsoft Corporation, Redmond, WA, USA).

### 2.11. Inhibition of Heat-Induced Protein Denaturation

The ability of the tested compounds to inhibit heat-induced protein denaturation was assessed using a modified version of a previously reported method [37]. Briefly, a 3.5% (*w*/*v*) bovine serum albumin (BSA) solution served as the protein substrate. The reaction mixture included 2.4 mL of BSA solution and 0.1 mL of the tested compound dissolved in dimethyl sulfoxide (DMSO) at concentrations ranging from 0.01 to 1 mM. For the control, 0.1 mL of DMSO was used instead of the test compound. The pH of the mixture was adjusted to 6.3 with 1 N HCl. The samples were first incubated at 37 °C for 20 min, then heated at 71 °C for 5 min to induce protein denaturation. After cooling to room temperature, 2.5 mL of phosphate-buffered saline (PBS, pH 6.3) was added to each sample. Absorbance was measured at 660 nm using a UV–Vis spectrophotometer. The percentage inhibition of protein denaturation was calculated using the following Equation (5):

(5) $$Inhibition\left(\%\right)=\left(1-\frac{A_{sample}}{A_{control}}\right)\times 100$$

where A_sample_ represents the absorbance of the tested sample, and A_control_ represents the absorbance of the control.

### 2.12. Evaluation of Antimicrobial Activity by Disk Diffusion Assay

Antimicrobial activity of DHCA, myrtenol, and myrtenyl dihydrocaffeate was evaluated using the agar disk diffusion method. Test compounds were dissolved in ethanol to create 100 mM stock solutions, and 10 µL of each was applied to sterile 6 mm paper disks (corresponding to 1.0 µmol per disk; 0.182 mg DHCA, 0.152 mg myrtenol, and 0.316 mg myrtenyl dihydrocaffeate per disk). Bacterial suspensions adjusted to 0.5 McFarland (~1.5 × 10^8^ CFU/mL) were evenly spread on Mueller–Hinton agar plates, with the impregnated disks placed on the inoculated surface. Plates were incubated at 37 °C for 16–18 h. After incubation, inhibition zone diameters were measured in millimeters (mm). A zone diameter of 6.0 mm was regarded as no inhibition beyond the disk. The following strains from the Polish Collection of Microorganisms (PCM, Institute of Immunology and Experimental Therapy, Polish Academy of Sciences, Wrocław, Poland) were tested: *Bacillus cereus* PCM 482, *Bacillus subtilis* PCM 486, *Enterobacter cloacae* PCM 2848, *Enterococcus faecalis* PCM 2909, *Escherichia coli* PCM 2057, *Klebsiella pneumoniae* PCM 1, *Listeria monocytogenes* PCM 2191, *Serratia marcescens* PCM 549, and *Staphylococcus aureus* PCM 2054.

### 2.13. Determination of Minimum Inhibitory and Bactericidal Concentrations (MIC/MBC) by Broth Microdilution

Minimum inhibitory concentrations (MICs) of DHCA, myrtenol, and myrtenyl dihydrocaffeate were determined using the broth microdilution method in sterile 96-well microplates. Stock solutions of test compounds were prepared in ethanol and diluted in Mueller–Hinton broth to reach concentrations up to 100 mM (two-fold serial dilutions). After inoculation, plates were incubated at 37 °C for 16–18 h. The MIC was recorded as the lowest concentration at which no visible growth was observed compared to the growth control. Minimum bactericidal concentrations (MBCs) were identified from wells with no visible growth, i.e., 3 µL from each clear well was plated onto Mueller–Hinton agar and incubated at 37 °C for 24 h. MBC was defined as the lowest concentration that resulted in no colony growth on the agar.

### 2.14. Statistical Analysis

Statistical analyses were performed using Statistica 13.3 (TIBCO Software Inc., Palo Alto, CA, USA). The results were analyzed by one-way analysis of variance (ANOVA) followed by Tukey’s HSD post hoc test, with statistical significance set at α = 0.05. In addition, the central composite design (CCD) and response surface modeling used to optimize the enzymatic esterification conditions were implemented in Statistica 13.3.

## 3. Results and Discussion

### 3.1. Optimization of Myrtenyl Dihydrocaffeate Synthesis

The reaction yield for the CALB-catalyzed esterification of DHCA with myrtenol was modeled and optimized through a four-factor central composite design. Table 2 summarizes the CCD experimental matrix along with observed yields, predicted values, and residuals. Across 18 runs, the yield ranged from 3.32 percent (Experiment 11) to 42.11 percent (Experiment 15), indicating a strong dependence on reaction conditions and supporting the use of response-surface modeling. It should be noted that the highest isolated yield observed in Experiment 15 (42.11%) was calculated relative to myrtenol, the limiting reagent at the myrtenol:DHCA ratio of 0.32:1. Therefore, this value should not be interpreted as a higher product output relative to DHCA. When expressed relative to the initial DHCA amount, the yield in Experiment 15 corresponds to approximately 13.48%.

Comparison of candidate models (Table 3) revealed a steady improvement in fit from linear to higher-order models. The most accurate was the full quadratic model (R^2^ = 0.9956; adjusted R^2^ = 0.9751), which was selected for further interpretation and optimization. Figure S1 illustrates the overall agreement between observed and fitted values, showing points closely aligned with the identity line. This is consistent with the high R^2^ and small residuals reported in Table 2, with the largest deviations seen at the replicated center points in Experiments 17–18.

**Figure 2:**
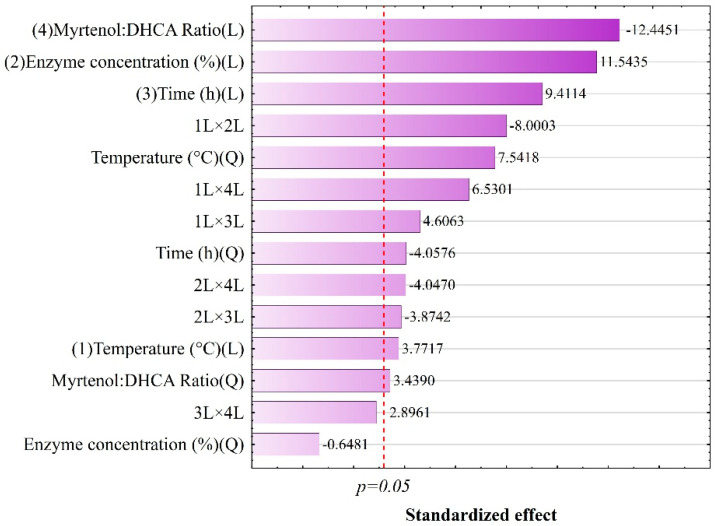
Pareto chart of standardized effects for the central composite design (CCD), with the response being reaction yield (%). Factors: 1—incubation temperature (°C), 2—enzyme concentration (%), 3—time (h), 4—myrtenol:DHCA ratio. L—linear (first-order) term; Q—quadratic (squared) term; and 1 L × 2 L denotes the two-factor interaction between the linear effects of factors 1 and 2 (analogously for other pairs). The vertical red dashed line indicates the critical value for significance at α = 0.05; effects to the right are statistically significant.

The standardized-effect Pareto chart (Figure 2) identifies terms that surpass the significance threshold (α = 0.05). This aligns with the ANOVA results for the full quadratic model (Table S1), which show significant effects for the linear factors of temperature, enzyme concentration, time, and the myrtenol:DHCA ratio, along with several quadratic effects, particularly temperature, time, and the ratio. Multiple two-factor interactions also proved significant, including temperature × enzyme concentration (1 L × 2 L), temperature × time (1 L × 3 L), temperature × ratio (1 L × 4 L), enzyme concentration × time (2 L × 3 L), and enzyme concentration × ratio (2 L × 4 L), all with *p* < 0.05 (Table S1). Conversely, the interaction between time and ratio (3 L × 4 L) was not statistically significant at α = 0.05, with a *p*-value of 0.0627 (Table S1).

**Figure 3:**
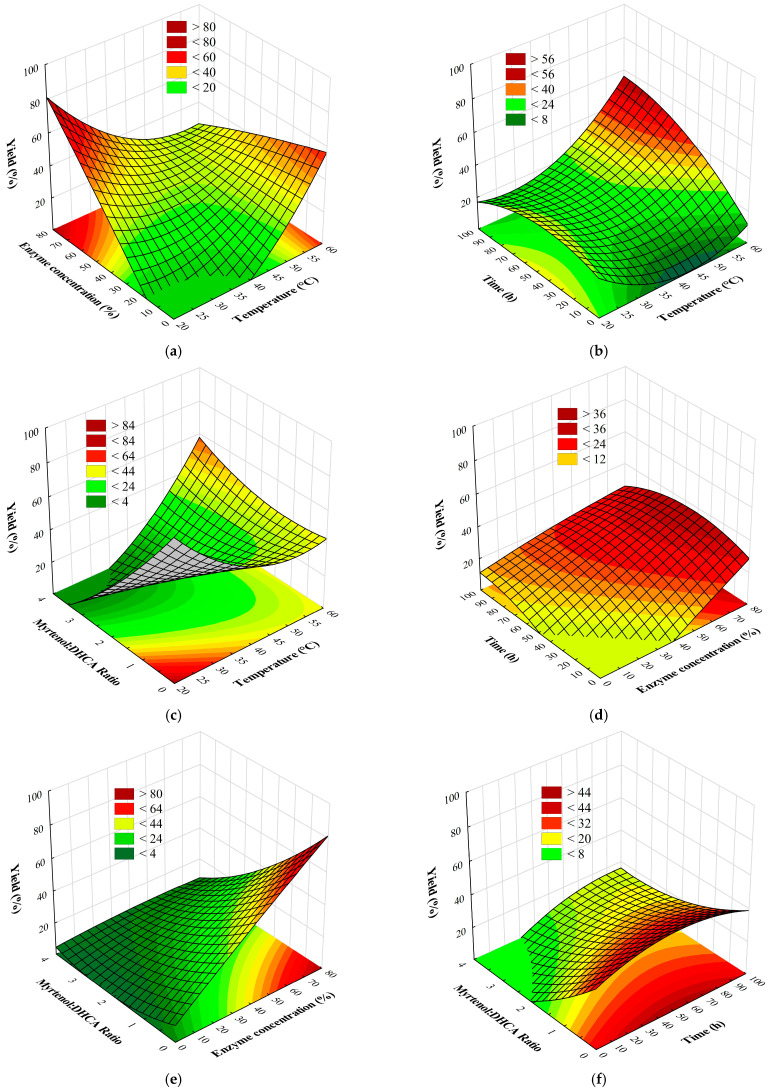
Three-dimensional response-surface plots predicted by the full quadratic model for reaction yield (%): (**a**) temperature vs. enzyme concentration, (**b**) temperature vs. time, (**c**) temperature vs. myrtenol:DHCA ratio, (**d**) enzyme concentration vs. time, (**e**) enzyme concentration vs. myrtenol:DHCA ratio, and (**f**) time vs. myrtenol:DHCA ratio.

**Figure 4:**
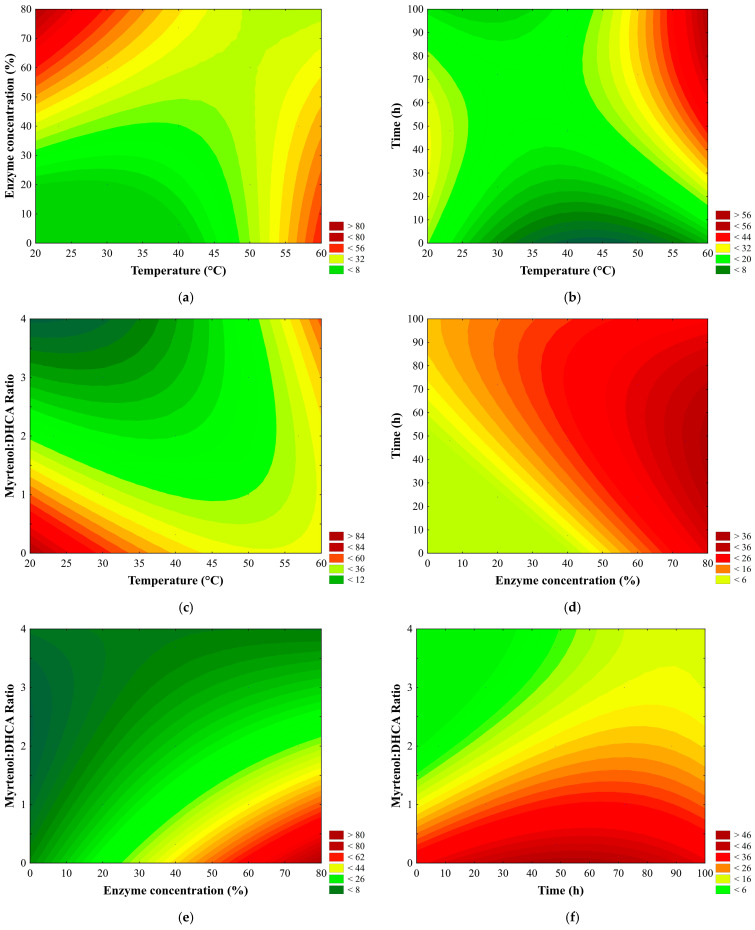
Contour plots of the predicted reaction yield (%) from the full quadratic model for reaction yield (%): (**a**) temperature vs. enzyme concentration, (**b**) temperature vs. time, (**c**) temperature vs. myrtenol:DHCA ratio, (**d**) enzyme concentration vs. time, (**e**) enzyme concentration vs. myrtenol:DHCA ratio, and (**f**) time vs. myrtenol:DHCA ratio.

The impact of factor pairs on yield is shown through 3D response surfaces (Figure 3a–f) and their contour plots (Figure 4a–f). These visuals highlight significant quadratic curvature and interactions, especially between the myrtenol:DHCA ratio and enzyme concentration, indicating that the yield landscape is not simply additive. Variations in one factor can significantly influence the effect of another.

The desirable conditions based on the full quadratic model are shown in Table 4, with predicted optimal parameters including a temperature of 56.818 °C, enzyme concentration of 56.818% (*w*/*w*, relative to DHCA weight), a processing time of 88.363 h, and a myrtenol:DHCA ratio of 3.6818:1. Under these conditions, the model projected a yield of 46.8195%, with a 95% prediction interval of 25.4039–68.2351% (Table 4).

Experimental validation at these settings yielded 39.94 ± 1.16%, corresponding to 85.31% of the predicted value and a 2.90% RSD. The experimental mean falls within the model’s 95% prediction interval, demonstrating consistency between the model’s predictions and actual results.

The moderate isolated yield obtained under the validated optimum may partly reflect the thermodynamic limitations of direct esterification. Lipase-catalyzed esterification is reversible, and water generated as a by-product may shift the reaction equilibrium toward hydrolysis, especially when no water-scavenging agent is used. In the present study, MTBE was used as the fixed reaction medium, and no molecular sieves were added. Therefore, the system’s initial water activity and the possible accumulation of water during the reaction were not independently controlled. The selection of MTBE was supported by previous solvent-screening studies with CALB, which showed that enzyme activity depends strongly on the organic medium. That substrate solubility is an important criterion in solvent selection. In that study, MTBE was among the solvent systems that maintained favorable CALB activity, together with acetone, *tert*-butanol, acetonitrile, and hydrophobic hydrocarbons [23]. Nevertheless, solvent type, solvent drying, controlled water activity, and the use of activated molecular sieves should be considered in future optimization efforts to further improve myrtenyl dihydrocaffeate synthesis.

The usefulness of response surface methodology (RSM) in the present work aligns with previous studies on lipase-catalyzed esterification and transesterification, in which product formation was governed by the simultaneous effects of temperature, enzyme loading, reaction time, substrate molar ratio, and reaction medium. Chen et al. [38] applied RSM to optimize the CALB-catalyzed synthesis of caffeic acid phenethyl ester, showing that reaction time, substrate molar ratio, and process conditions significantly affected ester conversion. Similarly, Gholivand et al. [39] used RSM to study the enzymatic synthesis of hexyl dihydrocaffeate and identified the alcohol-to-DHCA molar ratio as the most important factor affecting esterification yield.

These observations align with the present results, in which the myrtenol:DHCA molar ratio, enzyme concentration, reaction time, and temperature significantly affected myrtenyl dihydrocaffeate formation. The presence of significant linear and quadratic terms, along with several factor interactions, confirms that the reaction system did not respond simply to individual variables. Instead, the esterification yield depended on the combined balance among substrate availability, enzyme activity, mass transfer, water activity, and reaction equilibrium, all of which are recognized as key factors governing lipase-catalyzed esterification processes [40].

The optimized experimental yield in this study (39.94 ± 1.16%) should also be considered in light of the substrates’ structural characteristics. DHCA contains a polar catechol moiety and a carboxylic group, whereas myrtenol is a hydrophobic bicyclic monoterpene alcohol. This polarity mismatch may limit substrate accessibility in the reaction medium and reduce the efficiency of productive enzyme–substrate interactions. Similar challenges have been reported in the enzymatic lipophilization of phenolic acids, where increasing substrate hydrophobicity improved the applicability of the resulting esters in lipid-based systems but often required careful optimization of reaction conditions to achieve satisfactory conversion [38,39,40].

### 3.2. In Silico Predictions

#### 3.2.1. ADMET Properties

The ADMET properties of Myrtenol, DHCA, and the synthesized Myrtenyl dihydrocaffeate ester are summarized in Table S2. Because these parameters were derived using computational tools and have not been experimentally validated, they should be interpreted as preliminary indicators rather than definitive pharmacokinetic or toxicological evidence. The ester showed high predicted human intestinal absorption (93.885%), comparable to Myrtenol (94.799%) and higher than DHCA (55.476%). The ester exhibited lower water solubility (−3.491 log mol/L) but maintained favorable membrane permeability, with Caco-2 permeability of 1.078 log Papp and skin permeability of −3.122 log Kp.

For the distribution profile, the Myrtenyl dihydrocaffeate ester also exhibited lower blood–brain barrier permeability (−0.366 log BB) than Myrtenol (0.769), indicating potentially reduced central nervous system exposure. Additionally, the ester had a predicted stronger plasma protein binding profile (a fraction unbound value of 0.103) relative to Myrtenol (0.496) and DHCA (0.364).

In the metabolic prediction profile, the synthesized ester was predicted to be an unlikely inhibitor or substrate of the major CYP450 enzymes evaluated—CYP2D6 and CYP3A4 -, indicating low potential for CYP-mediated drug interactions. The toxicity assessment further revealed that the ester was unlikely to be mutagenic (AMES-negative) and hepatotoxic. It was also predicted to be non-sensitizing to the skin, unlike Myrtenol. The predicted oral acute toxicity LD_50_ value was higher for the ester (2.328 mol/kg) for the ester relative to Myrtenol (1.805 mol/kg) and DHCA (1.995 mol/kg). This indicated a potential lower toxicity for the ester.

Inhibition of hERG I and hERG II is considered an indicator of possible cardiotoxic liability. The synthesized ester was predicted not to inhibit hERG I but to inhibit hERG II, whereas both parent compounds, DHCA and myrtenol, were predicted to be non-inhibitors of hERG II (Table S2). This difference suggests that the hERG II alert may be associated with structural changes introduced by esterification, particularly increased lipophilicity and the combination of the aromatic catechol-containing DHCA moiety with the hydrophobic bicyclic myrtenyl fragment. These features may increase the likelihood of interaction with hydrophobic binding regions considered in the prediction model. However, the predicted hERG II inhibition should be interpreted cautiously, as it represents only an in silico alert and was not experimentally verified. Nevertheless, this result suggests that potential systemic safety limitations should be considered in future studies, particularly if oral or systemic applications are contemplated. Therefore, the current computational safety profile supports a more cautious interpretation of myrtenyl dihydrocaffeate as a candidate for lipid-based, cosmetic, or topical formulations rather than as an orally administered drug-like compound.

Overall, the synthesized ester showed favorable predicted permeability, an acceptable metabolic profile, and no predicted hepatotoxicity or skin sensitization. However, these ADMET results are computational predictions only and should be considered preliminary until experimentally validated.

**Figure 5:**
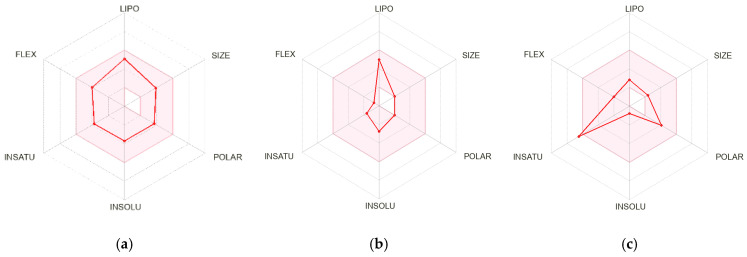
Drug-likeness/bioavailability radar plots of (**a**) Myrtenyl dihydrocaffeate, (**b**) Myrtenol, and (**c**) DHCA based on key physicochemical parameters.

The bioavailability radar plots (Figure 5) revealed variations in the drug-likeness profiles of the investigated compounds based on six key physicochemical parameters: lipophilicity, size, polarity, solubility, flexibility, and saturation. The synthesized ester, Myrtenyl dihydrocaffeate, has a relatively balanced profile across the six parameters—all the parameters fell within the normal/optimal pink region of the radar plot, while the parent molecules, Myrtenol and DHCA, showed slight deviations in lipophilicity, polarity, and saturation. This suggests a favorable oral bioavailability and drug-likeness potential.

#### 3.2.2. Bioactivity Spectrum

The predicted bioactivity spectrum of the synthesized myrtenyl dihydrocaffeate ester is presented in Table 5 and Table S3. The result revealed several potential biological activities of the ester. This includes mucomembranous protective (Pa = 0.719) with the highest probability; cholesterol antagonistic (Pa = 0.645), phosphatase inhibitory (Pa = 0.637), and anti-inflammatory activities (Pa = 0.632). The ester also demonstrated predicted antithrombotic (Pa = 0.606), MMP9 expression inhibitory (Pa = 0.604), and fibrinolytic (Pa = 0.600) activities. Notably, the parent compounds myrtenol and DHCA are reported to have strong antioxidant and anti-inflammatory activities [13,41,42,43,44,45], thus these activities were considered particularly relevant to the ester and investigated further.

#### 3.2.3. Predicted Proteins and Molecular Docking Simulation

**Figure 6:**
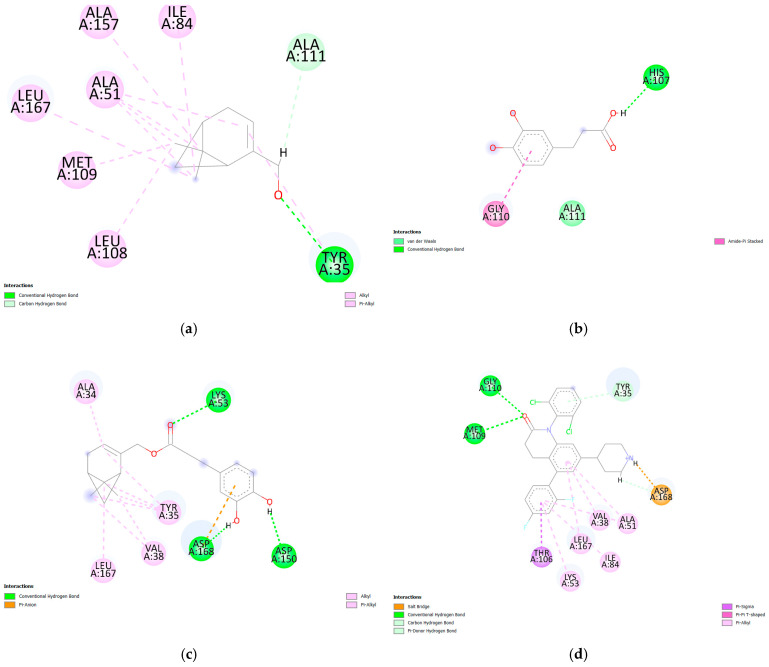
Post-docking compound-protein interactions (**a**) Myrtenol (**b**) DHCA (**c**) Myrtenyl dihydrocaffeate (**d**) Control (PubChem ID: 447725) in complex with p38 MAP kinase (1OVE).

**Figure 7:**
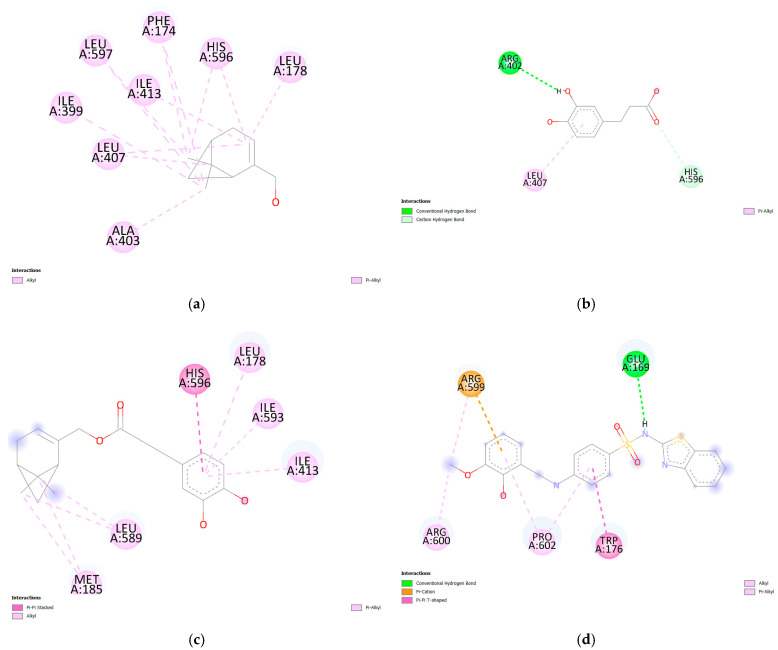
Post-docking compound-protein interactions (**a**) Myrtenol (**b**) DHCA (**c**) Myrtenyl dihydrocaffeate (**d**) Control (PubChem ID: 70701426) in complex with Human 12-lipoxygenase (8HGZ).

**Figure 8:**
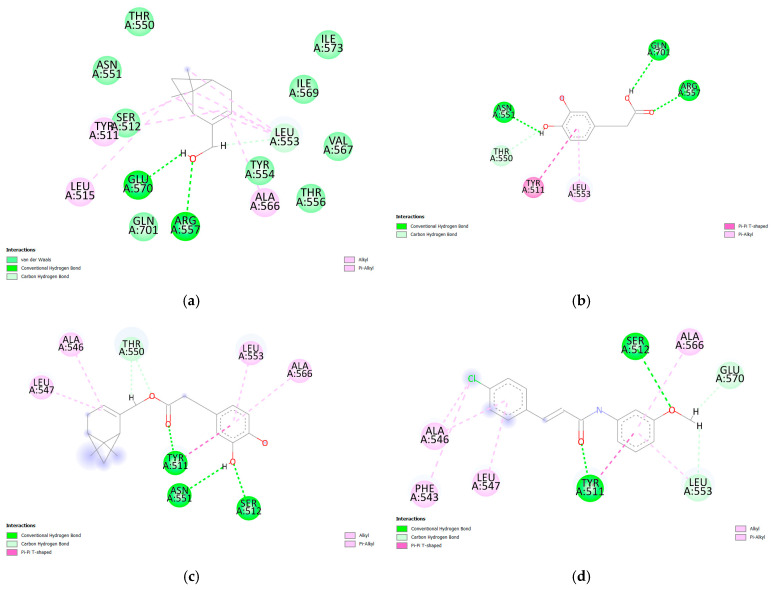
Post-docking compound-protein interactions (**a**) Myrtenol (**b**) DHCA (**c**) Myrtenyl dihydrocaffeate (**d**) Control (PubChem ID: 667594) in complex with Transient receptor potential cation channel subfamily V member 1 (8GFA).

Several proteins were predicted for the synthesized ester (Table S4), however, with low probability scores for both databases. This may be due to the myrtenyl moiety not being a very common compound with many known activities, unlike the phenolic pharmacophore. Proteins relevant to the predicted anti-inflammatory bioactivity were selected, and the synthesized ester and its parent compounds were docked against these proteins. The result is presented in Table 6 and Figure 6, Figure 7 and Figure 8.

In the docking simulation against p38 MAPK, the synthesized ester showed an SILE value of 2.37, which was comparable to DHCA (2.36) and slightly higher than myrtenol (2.27), but lower than the control compound (3.72). Despite the lower score relative to the control, the ester exhibited interesting multiple interactions with the active site amino acid residues, including conventional hydrogen bonds with Lys53, Asp150, and Asp168 [46], comparable to the control, which equally had interesting interactions with important active site amino acid residues like -Asp 168, Met 109, Gly 110 [46].

In the docked complexes with human 12-lipoxygenase (12-LOX) as target, the ester exhibited an SILE value of 2.65, which was slightly higher than the control (2.55) and the parent compounds (DHCA and Myrtenol) with SILE values of 2.55 and 2.16, respectively. The interaction analysis revealed a complex stabilized primarily by hydrophobic interactions, including Pi-Pi stacked interaction with His596 and Pi-alkyl interactions with Leu178, Ile413, and Ile593 involving important amino acid residues within the lipophilic active pocket of lipoxygenases [47]. Although DHCA formed hydrogen bonds with Arg402 and His596, the ester demonstrated strong aromatic and hydrophobic interactions.

For Transient Receptor Potential Vanilloid 1 (TRPV1), the ester showed an SILE value of 2.54, comparable to that of the control compound (2.55). Although DHCA exhibited a slightly higher value (2.75), the ester performed substantially better than myrtenol (1.64), indicating that esterification improved myrtenol’s interaction potential. The interaction map revealed hydrogen bond interactions with Important active site residues-Tyr511, Ser512, and Asn551 [48], as well as hydrophobic interactions with Ala546, Leu547, Leu553, and Ala566. Importantly, a similar binding pattern and interaction profile were also observed in the complex with the control ligand, suggesting a similar binding pose and potential modulatory activity with the ester.

Overall, these results revealed comparable bioactivity and improved druglikeness and ADMET properties with the ester (except for hERG II Inhibition). The observed hERG inhibition, as predicted, may raise concerns for systemic/oral drug development; the ester may be very useful as an anti-inflammatory topical/skincare agent.

The predicted ADMET and docking profiles in this study align with the general principle that esterifying phenolic compounds can enhance their compatibility with hydrophobic environments while preserving key structural features responsible for biological activity. Phenolipids are commonly described as amphiphilic phenolic derivatives, and lipophilization improves interactions with oils, emulsions, micelles, liposomes, and biological membranes, thereby broadening their applicability in food, cosmetic, and biomedical systems [49]. Similar trends were recently reported for enzymatically esterified mangiferin derivatives, where esterification increased liposolubility by 10–30-fold and improved antioxidant performance in lipid systems while largely preserving biological activity [50].

In the present study, myrtenyl dihydrocaffeate exhibited higher predicted intestinal absorption and favorable membrane permeability than DHCA, which may be attributable to the hydrophobic myrtenyl moiety. This aligns with reports on esterified polyphenols, including EGCG derivatives, in which introducing hydrophobic groups improved lipid solubility, stability, and potential bioavailability [51]. Moreover, studies on phenolic esters have shown that ester structure can influence hydrolysis, controlled release of parent phenolic compounds, and absorption kinetics, suggesting that esterification may substantially modify the pharmacokinetic behavior of phenolic molecules [52].

The molecular docking results should be interpreted with particular caution. The protein targets selected for docking were identified using computational target prediction tools and had low probability scores. Therefore, MAPK14, ALOX12, and TRPV1 should be regarded only as hypothetical targets of myrtenyl dihydrocaffeate. The docking results may suggest that the ester can form stabilizing interactions within the selected protein binding pockets, with the DHCA-derived catechol moiety contributing to hydrogen bonding and aromatic interactions, while the myrtenyl fragment may support hydrophobic contacts. However, these observations do not confirm biological target engagement or anti-inflammatory activity. Experimental enzyme inhibition, receptor binding, or cell-based assays would be required to validate any of these proposed interactions. Accordingly, the present in silico results should be treated as hypothesis-generating and used only to guide future mechanistic studies. Based on the current experimental evidence, the most strongly supported functionality of myrtenyl dihydrocaffeate is its antioxidant performance and its ability to improve oxidative stability in a lipid matrix. Its possible interaction with inflammation-related targets remains speculative and requires validation.

### 3.3. Antioxidant Activity

Dihydrocaffeic acid (DHCA) is a phenolic compound recognized for its antioxidant activity. Thus, an antioxidant screening was performed to compare DHCA with its lipophilic derivative, myrtenyl dihydrocaffeate, and a standard antioxidant, BHT. The IC_50_ values obtained from the ABTS and DPPH radical scavenging assays are shown in Table 7. In the ABTS assay, all three compounds exhibited similar activity, with IC_50_ values ranging from approximately 1.14 to 1.25 mM, and no statistically significant differences were detected among them (Table 7). In contrast, the DPPH assay provided a clearer distinction: DHCA showed the highest scavenging activity (IC_50_ = 0.11 mM), myrtenyl dihydrocaffeate had slightly lower activity (IC_50_ = 0.16 mM), and BHT was considerably less effective (IC_50_ = 0.65 mM), with significant differences between groups (*p* < 0.05, Table 7).

The results of the present study are consistent with previous investigations of lipophilic derivatives of dihydrocaffeic acid. In an earlier study, butyl dihydrocaffeate exhibited DPPH radical-scavenging activity with an IC_50_ of 0.16 mM, compared with 0.12 mM for the parent DHCA, indicating that esterification slightly reduced but largely preserved the antioxidant capacity associated with the catechol moiety. Similar observations were made in the current work, where myrtenyl dihydrocaffeate showed only a moderate decrease in DPPH-scavenging activity relative to DHCA (0.16 vs. 0.11 mM), while remaining substantially more active than BHT. These findings support the hypothesis that the catechol ring remains the primary structural element responsible for radical-scavenging activity, whereas modification of the carboxyl group mainly affects physicochemical properties rather than the antioxidant mechanism itself [14].

A similar trend was observed in a more recent study of a homologous series of alkyl dihydrocaffeates (C4–C12), in which DPPH activity gradually decreased with increasing alkyl chain length, yet all esters retained strong antioxidant properties. The butyl derivative exhibited an IC_50_ of 0.14 mM, very close to that of DHCA, indicating that lipophilization does not necessarily cause a substantial loss of radical-scavenging activity. The results for myrtenyl dihydrocaffeate further extend these observations and suggest that not only alkyl chain length but also the structure of the alcohol moiety may influence antioxidant effectiveness [35].

These observations are consistent with the general concept of phenolipids, in which esterification increases lipophilicity while preserving the antioxidant activity of the phenolic core [12,49].

### 3.4. Oxidative Stability of Rapeseed Oil

Rapeseed oil used in this study was characterized by a high proportion of unsaturated fatty acids (92.55%), with oleic acid (C18:1, 62.09 ± 0.45%) as the dominant fatty acid, followed by linoleic acid (C18:2, 19.84 ± 0.06%) and α-linolenic acid (C18:3, 8.95 ± 0.25%) (Table S5). The total MUFA and PUFA contents were 63.77 ± 0.48% and 28.78 ± 0.18%, respectively, whereas saturated fatty acids accounted for only 7.46 ± 0.29%. This fatty acid profile is nutritionally favorable but also makes the oil susceptible to oxidative deterioration, particularly because of its polyunsaturated fatty acids.

The oxidative stability of rapeseed oil supplemented with DHCA, myrtenol, and myrtenyl dihydrocaffeate was evaluated by PDSC at temperatures from 110 to 140 °C (Table 8). In all cases, oxidation time decreased with increasing temperature, reflecting the acceleration of lipid oxidation at higher temperatures. The addition of DHCA and myrtenyl dihydrocaffeate significantly prolonged oxidation times relative to the control sample across all tested temperatures (*p* < 0.05). At 120 °C, the oxidation time increased from 79.05 min for the control oil to 119.61 min for DHCA and 116.22 min for myrtenyl dihydrocaffeate, respectively. Similar trends were observed at the remaining temperatures. Protection factors ranged from 1.32 to 1.51 for DHCA and from 1.32 to 1.47 for the ester, indicating substantial improvement in oxidative stability. In contrast, myrtenol exhibited only a marginal protective effect, with protection factors close to unity (0.94–1.06), indicating little to no protective effect on rapeseed oil under the PDSC conditions used. Although myrtenol contains an allylic alcohol moiety, it lacks phenolic hydroxyl groups capable of efficient hydrogen atom donation or resonance stabilization of the resulting radical. Therefore, its radical-scavenging and chain-breaking antioxidant capacity in lipid oxidation is expected to be much weaker than that of DHCA and myrtenyl dihydrocaffeate, both of which contain a catechol structure. Moreover, under accelerated oxidation conditions, volatile monoterpene alcohols such as myrtenol may be partially lost or transformed, further limiting their ability to protect the oil phase. Thus, the lack of a marked protective effect of myrtenol is consistent with the dominant role of the DHCA-derived catechol moiety in delaying lipid oxidation.

The Arrhenius model provided an excellent description of the oxidation process, as indicated by the high coefficients of determination (R^2^ = 0.9975–0.9990; Table 9). All additives increased the activation energy of oxidation relative to the control oil, indicating a higher energetic barrier to the initiation of oxidation. However, practical antioxidant performance was better reflected by oxidation times and calculated reaction rate coefficients. Both DHCA and myrtenyl dihydrocaffeate consistently reduced oxidation rates across the temperature range investigated, whereas myrtenol showed only negligible effects.

The calculated reaction rate coefficients are presented in Supplementary Table S6. Consistent with the oxidation time data, both DHCA and myrtenyl dihydrocaffeate reduced the oxidation rate of rapeseed oil at all tested temperatures. At 120 °C, the reaction rate coefficient decreased from 0.012509 min^−1^ in the control sample to 0.008847 min^−1^ and 0.008957 min^−1^ in the presence of DHCA and myrtenyl dihydrocaffeate, respectively. Similar reductions were observed at 110, 130, and 140 °C. Overall, DHCA and myrtenyl dihydrocaffeate reduced oxidation rates by approximately 26–31% and 26–30%, respectively, compared with the control oil. In contrast, myrtenol exerted only a negligible effect on oxidation kinetics, with k values remaining close to those of the control sample across the temperature range investigated.

The obtained results are consistent with the antioxidant mechanism of catechol-containing compounds. The presence of two adjacent hydroxyl groups in the aromatic ring enables efficient scavenging of lipid-derived radicals and stabilization of the resulting phenoxyl radicals. Consequently, both DHCA and its ester effectively delayed oxidation, whereas myrtenol, lacking a phenolic structure, exhibited only limited antioxidant activity. The similar performance of DHCA and myrtenyl dihydrocaffeate further indicates that esterification of the carboxyl group did not substantially impair the radical-scavenging properties associated with the catechol moiety.

These observations align with the previous studies on alkyl dihydrocaffeates, which showed that both DHCA and its lipophilic derivatives significantly improved the oxidative stability of vegetable oils under accelerated oxidation conditions [35]. In that study, the effectiveness of DHCA esters varied with alkyl chain length and the oxidation system used, with longer-chain derivatives often performing better in lipid-rich environments. Similarly, the present results show that lipophilizing DHCA preserves its antioxidant effectiveness in bulk oil systems. Notably, myrtenyl dihydrocaffeate exhibited protection factors nearly identical to those of the parent acid, despite its increased lipophilicity, suggesting that introducing the terpene moiety did not compromise antioxidant performance. This observation is consistent with the general concept of phenolipids, where esterification improves compatibility with hydrophobic matrices while maintaining the antioxidant activity associated with the phenolic core structure [12,49].

### 3.5. Heat-Induced Protein Denaturation Assay

The heat-induced protein denaturation assay was used solely as a preliminary screening model to assess potential interactions between the tested compounds and a protein system. The full dataset is provided in Table S7. The tested compounds showed only weak apparent inhibition of protein denaturation. Among them, myrtenyl dihydrocaffeate exhibited the highest apparent inhibition across all tested concentrations, reaching a maximum of 14.94% at 0.50 mM. This value was significantly higher than those for DHCA and myrtenol at the same concentration (*p* < 0.05). However, the absolute level of inhibition remained low and should not be interpreted as evidence of strong anti-inflammatory activity.

Dihydrocaffeic acid showed a maximum apparent inhibition of 6.51% at 0.25 mM, whereas myrtenol reached 5.57% at 0.50 mM. Across all tested compounds, the response was non-monotonic, with apparent inhibition decreasing at 1.00 mM compared with lower concentrations. For myrtenyl dihydrocaffeate, inhibition decreased from 14.94% at 0.50 mM to 10.39% at 1.00 mM. This behavior indicates that the effect was not simply concentration-dependent and may reflect limited compound solubility, compound–protein interactions, or spectrophotometric interference at 660 nm due to turbidity, particularly for the more lipophilic ester.

Although esterification of DHCA with myrtenol increased the apparent inhibition of heat-induced protein denaturation compared with the parent compounds, the overall effect remained weak. At 0.50 mM, myrtenyl dihydrocaffeate showed approximately 2.3-fold higher apparent inhibition than DHCA and 2.7-fold higher than myrtenol. This may suggest a stronger interaction of the ester with the protein system used in this assay, possibly due to the combined presence of the catechol moiety and the hydrophobic myrtenyl fragment. Nevertheless, this interpretation should be treated with caution because the assay does not identify a specific inflammatory target or mechanism.

An important limitation of this experiment is the absence of a reference anti-inflammatory drug as a positive control and the lack of compound-specific turbidity blanks. Therefore, the present data should be regarded only as preliminary results for protein denaturation screening. They do not confirm anti-inflammatory activity. Further studies using appropriate positive controls, compound blanks, and validated biochemical or cell-based inflammatory models would be required to assess the anti-inflammatory relevance of myrtenyl dihydrocaffeate.

### 3.6. Antimicrobial Activity

**Figure 9:**
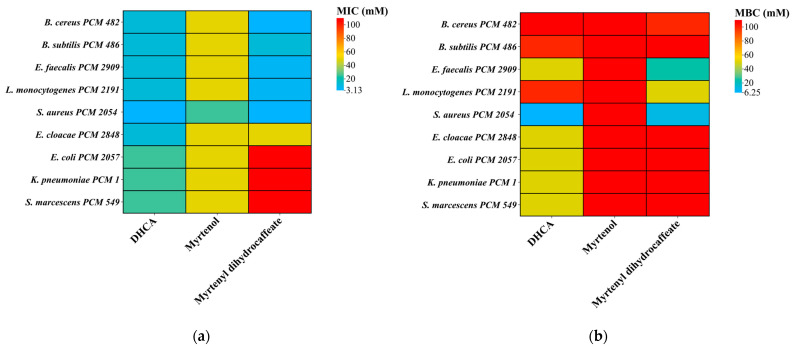
Heatmap visualization of antimicrobial activity (mM) of DHCA, myrtenol, and myrtenyl dihydrocaffeate against the tested bacterial strains: (**a**) minimum inhibitory concentration (MIC) and (**b**) minimum bactericidal concentration (MBC).

Despite the emphasis on antioxidant performance, phenolic acids are also often studied for their antimicrobial potential. Therefore, the antimicrobial activity of DHCA, myrtenol, and myrtenyl dihydrocaffeate was evaluated using a two-step process, i.e., an initial disk diffusion screening (Table 10), followed by quantitative MIC/MBC measurements (Figure 9).

Growth inhibition zones are shown in Table 10. For most strains, DHCA and myrtenol did not produce measurable inhibition zones, indicating no detectable inhibition beyond the disk diameter (6.0 ± 0.0 mm). An exception was *S. aureus* PCM 2054, where DHCA resulted in a clear inhibition zone measuring (12.3 ± 1.0 mm).

Unlike other compounds, myrtenyl dihydrocaffeate exhibited notable activity against several Gram-positive bacteria, with inhibition zones notably larger than those caused by DHCA and myrtenol for *B. cereus* PCM 482 (12.8 ± 0.5 mm), *B. subtilis* PCM 486 (11.8 ± 1.0 mm), *E. faecalis* PCM 2909 (11.0 ± 0.8 mm), and *L. monocytogenes* PCM 2191 (10.0 ± 0.8 mm) (Table 10). The strongest response was observed against *S. aureus* PCM 2054 (13.5 ± 0.6 mm), where the ester and DHCA produced similarly large inhibition zones, but myrtenol showed no activity. No inhibition zones larger than 6.0 mm were recorded for any of the tested Gram-negative bacteria (*E. cloacae*, *E. coli*, *K. pneumoniae*, *S. marcescens*) (Table 10).

These screening results, supported by MIC and MBC data and visualized through heatmaps (Figure 9), show that myrtenol had weak inhibitory effects (MIC of 25–50 mM) and no bactericidal activity within the tested range (MBC > 100 mM for all strains). DHCA demonstrated moderate growth inhibition, particularly against Gram-positive bacteria (MIC generally 12.5 mM, and 3.13 mM against *S. aureus*), while higher concentrations were needed for Gram-negative strains (MIC = 25 mM). The most significant improvement was observed with myrtenyl dihydrocaffeate, which exhibited lower MIC values against Gram-positive bacteria (e.g., 3.13 mM for *B. cereus* and *S. aureus*, 6.25 mM for *E. faecalis* and *L. monocytogenes*). This ester also showed bactericidal activity against certain Gram-positive strains (MBC = 25 mM for *E. faecalis* and 50 mM for *L. monocytogenes*), although Enterobacterales remained mostly resistant (MIC/MBC > 100 mM for *E. coli*, *K. pneumoniae*, and *S. marcescens*, while MIC 50 mM and MBC > 100 mM for *E. cloacae*). Overall, both Table 10 and Figure 9 demonstrate that the activity is primarily directed against Gram-positive bacteria, with limited effectiveness against Gram-negative strains.

Despite the improvement observed after esterification, the absolute MIC values indicate only weak antibacterial potency. For example, the MIC of myrtenyl dihydrocaffeate against *S. aureus* was 3.13 mM, approximately 1.0 mg/mL. Therefore, this result should not be interpreted as evidence of clinically relevant or therapeutic antimicrobial activity. Rather, the observed effect may be considered a mild supportive property, potentially relevant for lipid-based food systems, cosmetic formulations, or topical products, especially in combination with the antioxidant activity and the ability of myrtenyl dihydrocaffeate to improve oxidative stability in rapeseed oil. Further studies using formulation-based models, challenge tests, and broader microbial panels would be required to determine whether this weak but detectable antimicrobial effect has practical relevance in preservation-oriented applications.

The observed antimicrobial profile aligns with the general behavior of lipophilized phenolic compounds, whose activity often depends on the balance between the phenolic pharmacophore and the hydrophobic moiety. Phenolipids have been reported to interact more effectively with microbial membranes than their more hydrophilic parent compounds, and their antimicrobial activity is frequently associated with increased lipophilicity and improved affinity for lipid bilayers [49]. In the present study, esterification of DHCA with myrtenol clearly enhanced antibacterial activity against Gram-positive bacteria, whereas myrtenol alone remained weakly active or inactive. This suggests that the antimicrobial activity of myrtenyl dihydrocaffeate is not simply a consequence of the terpene alcohol fragment but rather results from the combined contributions of the catechol-containing DHCA moiety and the hydrophobic myrtenyl group.

These findings align with the previous study on butyl dihydrocaffeate, which found that the antimicrobial effect of lipophilization depended strongly on the microorganism tested [14]. In that work, butyl dihydrocaffeate showed MIC values against bacteria ranging from 4 to 16 mM, while DHCA showed MIC values ranging from 2 to 16 mM, indicating that esterification does not universally increase antibacterial potency but can modify the microbial selectivity profile [14]. In the present study, myrtenyl dihydrocaffeate showed a more pronounced activity against Gram-positive bacteria, particularly *B. cereus* and *S. aureus*, with MIC values up to 3.13 mM.

A similar structure-dependent antimicrobial behavior has been reported for other phenolic acid esters. Alkyl ferulates and alkyl gallates have shown chain-length-dependent antibacterial activity, with medium-chain derivatives often exhibiting the strongest effects against foodborne pathogens such as *E. coli*, *S. aureus*, and *L. monocytogenes* [53,54,55]. Proposed mechanisms include disruption of membrane integrity, changes in cell morphology, interference with DNA, and disturbance of respiratory electron transport, leading to oxidative stress in bacterial cells [53,54,55]. Although the mechanism of action of myrtenyl dihydrocaffeate was not directly investigated here, the ester’s higher activity relative to both parent compounds suggests that increased lipophilicity may facilitate interactions with bacterial envelopes.

The lack of activity against Gram-negative bacteria is also consistent with the known higher intrinsic resistance of Enterobacterales to many hydrophobic and phenolic antimicrobials. The outer membrane of Gram-negative bacteria serves as an additional permeability barrier, limiting the penetration of many compounds that more readily interact with the cytoplasmic membrane of Gram-positive bacteria [56]. This may explain why myrtenyl dihydrocaffeate was active mainly against *B. cereus*, *B. subtilis*, *E. faecalis*, *L. monocytogenes*, and *S. aureus*, while *E. coli*, *K. pneumoniae*, and *S. marcescens* remained resistant within the tested concentration range.

## 4. Conclusions

The present study demonstrated that myrtenyl dihydrocaffeate can be synthesized by CALB-catalyzed esterification of dihydrocaffeic acid with myrtenol under optimized reaction conditions established using response surface methodology. The developed biocatalytic process delivered satisfactory ester yields under mild reaction conditions consistent with green chemistry principles.

Lipophilization of DHCA produced a derivative that retained antioxidant activity and markedly improved the oxidative stability of rapeseed oil. PDSC analysis confirmed that myrtenyl dihydrocaffeate effectively delayed oxidation, yielding protection factors comparable to those of the parent phenolic acid. In contrast, myrtenol alone provided only a limited protective effect, underscoring the key role of the catechol moiety in antioxidant performance.

The synthesized ester also exhibited antimicrobial activity, particularly against selected Gram-positive bacteria, as confirmed by disk diffusion and MIC/MBC assays. However, this activity should be considered mild and more relevant to preservative or topical applications than to therapeutic antimicrobial use. The heat-induced protein denaturation assay showed only weak, non-monotonic apparent inhibition and should be considered a preliminary screen rather than evidence of anti-inflammatory activity. Similarly, the molecular docking and target prediction results should be regarded only as preliminary, hypothesis-generating observations, because the predicted protein targets showed low probability scores and were not validated by enzyme-level, receptor-level, or cell-based assays.

Overall, the findings demonstrate that esterifying DHCA with myrtenol is a promising strategy for producing lipophilic antioxidants with enhanced applicability in lipid-containing systems. Further studies should focus on optimizing the reaction medium and water activity, validating the proposed molecular targets experimentally, and evaluating the compound in more complex lipid-based food, cosmetic, and topical formulation models.

## Figures and Tables

**Table 1:** Central composite design (CCD) factor levels for the CALB-catalyzed esterification of DHCA with myrtenol, showing coded levels (−α, −1, 0, +1, +α) and corresponding actual values for temperature (A), enzyme concentration (B, % *w*/*w* relative to DHCA mass), reaction time (C, h), and molar ratio (D, myrtenol:DHCA).

Factor	Name	−α	−1	0	+1	+α
A	Temperature (°C)	23.18	30.00	40.00	50.00	56.82
B	Enzyme concentration (% *w*/*w*)	6.36	20.00	40.00	60.00	73.64
C	Time (h)	7.64	24.00	48.00	72.00	88.36
D	Ratio (myrtenol:DHCA)	0.32:1 *	1:1	2:1	3:1	3.68:1

* Note: At myrtenol:DHCA ratios ≥ 1:1, DHCA was the limiting reagent. At the lowest axial point of the molar ratio factor (0.32:1), myrtenol was the limiting reagent.

**Table 2:** Experimental matrix of the four-factor central composite design (CCD) used to model reaction yield.

Exp. No.	Temperature (°C)	Enzyme Concentration (%)	Time (h)	Myrtenol:DHCA Ratio	Yield (%)	Predicted Yield (%)	Residuals
1	50.00	60.00	72.00	1.00	37.79	37.97	−0.19
2	50.00	60.00	24.00	1.00	31.21	31.18	0.03
3	50.00	20.00	72.00	3.00	38.89	39.08	−0.19
4	30.00	60.00	24.00	3.00	15.79	15.75	0.03
5	50.00	20.00	24.00	3.00	14.05	14.01	0.03
6	30.00	20.00	72.00	1.00	17.43	17.62	−0.19
7	30.00	60.00	72.00	3.00	17.75	17.94	−0.19
8	30.00	20.00	24.00	1.00	12.83	12.80	0.03
9	23.18	40.00	48.00	2.00	26.69	26.58	0.11
10	56.82	40.00	48.00	2.00	36.87	36.76	0.11
11	40.00	6.36	48.00	2.00	3.32	3.21	0.11
12	40.00	73.64	48.00	2.00	34.47	34.36	0.11
13	40.00	40.00	7.64	2.00	5.09	5.25	−0.16
14	40.00	40.00	88.36	2.00	21.97	21.59	0.38
15	40.00	40.00	48.00	0.32 *	42.11	42.00	0.11
16	40.00	40.00	48.00	3.68	8.53	8.42	0.11
17	40.00	40.00	48.00	2.00	21.97	19.80	2.16
18	40.00	40.00	48.00	2.00	17.38	19.80	−2.42

* Note: Yield values were calculated relative to the theoretical ester yield based on the limiting reagent. DHCA was limiting in all experiments with myrtenol:DHCA ratios ≥ 1:1, whereas myrtenol was limiting in Experiment 15, performed at a ratio of 0.32:1.

**Table 3:** Comparison of candidate response-surface models fitted to the CCD data. Reported are R_2_ and adjusted R_2_ for four structures: Linear (main effects only), Pure Quadratic (no interactions), Two-Factor Interaction (2FI), and Full Quadratic (second-order; 2FI + squared terms).

Model	R_2_ *	R_2Adj_
Linear	0.5904	0.4644
Pure Quadratic	0.7496	0.5271
Two-Factor Interaction	0.8364	0.6026
Full Quadratic	0.9956	0.9751 (Suggested)

* R_2_—coefficient of determination, R_2Adj_—Adjusted R_2_ value.

**Table 4:** Predicted optimum conditions (desirability function) for CALB-catalyzed esterification of DHCA with myrtenol and validation results.

Parameter	Optimum (Predicted)
Temperature	56.818 °C
Enzyme concentration (relative to DHCA mass)	56.818% (*w*/*w*)
Reaction time	88.363 h
Molar ratio (myrtenol:DHCA)	3.6818:1
Predicted yield (Y_pred_)	46.8195%
95% prediction interval (PI) for yield	25.4039–68.2351%
Experimental yield at optimum (validation, Y_exp_)	39.94 ± 1.16%
Validation	85.31%
RSD	2.90%
Relative prediction error (based on mean)	14.69%

Y_exp_ is reported as mean ± SD. Validation (%) = (Y_exp_/Y_pred_) × 100. RSD (%) = (SD/Y_exp_) × 100. Relative prediction error (%) = ∣Y_exp_ − Y_pred_∣/Y_pred_ × 100.

**Table 5:** Predicted bioactivity spectrum of myrtenyl dihydrocaffeate.

Pa	Pi	Bioactivity
0.719	0.048	Mucomembranous protector
0.653	0.007	UGT1A4 substrate
0.645	0.014	Cholesterol antagonist
0.637	0.031	Phosphatase inhibitor
0.635	0.015	Analeptic
0.632	0.026	Antiinflammatory
0.629	0.020	Respiratory analeptic
0.628	0.018	UDP-glucuronosyltransferase substrate
0.606	0.015	Antithrombotic
0.604	0.015	MMP9 expression inhibitor
0.601	0.090	Testosterone 17beta-dehydrogenase (NADP^+^) inhibitor
0.600	0.073	Fibrinolytic

**Table 6:** Molecular docking scores, ligand efficiency, and binding interactions of myrtenol, DHCA, myrtenyl dihydrocaffeate ester, and control ligand against selected targets.

Target Protein (PDB ID)	Docking Score	SILE	Ligand/Complex	Amino Acid Residues and Interactions
p38 MAP kinase (1OVE)	−4.9702	2.36	Myrtenol	Tyr35 ^ag^, Ala111 ^b^, Ala51 ^h^, Ala157 ^h^, Leu108 ^h^, Met109 ^h^, Ile84 ^h^, Leu167 ^h^
	−5.1135	2.37	DHCA	His107 ^a^, Gly110/Ala111 ^k^
	−6.2367	2.37	Myrtenyl dihydrocaffeate	Lys53 ^a^, Asp150 ^a^, Asp168 ^ai^, Ala34 ^h^, Leu167 ^h^, Val38 ^h^, Tyr35 ^g^
	−9.6602	3.27	Control	Asp168 ^cb^, Met109 ^a^, Gly110 ^a^, Tyr35 ^df^, Thr106 ^e^, Val38 ^g^, Ala51 ^g^, Leu167 ^g^, Lys53 ^g^, Ile84 ^g^
Human 12-lipoxygenase (8HGZ)	−4.6146	2.19	Myrtenol	Ala403 ^h^, Leu407 ^h^, Leu178 ^h^, Ile413 ^h^, Leu597 ^h^, Ile399 ^h^, Phe174 ^g^, His596 ^g^
	−5.5019	2.55	DHCA	Arg402 ^a^, His596 ^b^, Leu407 ^g^
	−6.9506	2.65	Myrtenyl dihydrocaffeate	His596 ^l^, Met185 ^h^, Leu589 ^h^, Leu178 ^g^, Ile413 ^g^, Ile593 ^g^
	−6.9261	2.50	Control	Glu169 ^a^, Arg599 ^jh^, Trp176 ^f^, Arg600 ^h^, Pro602 ^g^
Transient receptor potential cation channel subfamily V member 1 (8GFA)	−3.4640	1.64	Myrtenol	Arg557 ^a^, Glu570 ^a^, Leu553 ^bh^, Ala566 ^h^, Leu515 ^h^, Tyr511 ^g^
	−5.9430	2.75	DHCA	Arg557 ^a^, Asn551 ^a^, Gln701 ^a^, Thr550 ^b^, Tyr511 ^f^, Leu553 ^g^
	−6.6793	2.54	Myrtenyl dihydrocaffeate	Tyr511 ^af^, Ser512 ^a^, Asn551 ^a^, Thr550 ^b^, Ala546 ^h^, Leu547 ^h^, Leu553 ^g^, Ala566 ^g^
	−6.3539	2.55	Control	Tyr511 ^af^, Ser512 ^a^, Glu570 ^b^, Leu553 ^bg^, Ala546 ^hg^, Phe543 ^g^, Leu547 ^g^, Ala566 ^g^

^a^ Conventional Hydrogen Bond, ^b^ Carbon Hydrogen Bond, ^c^ Salt Bridge/Attractive Charge, ^d^ Pi-Donor Hydrogen Bond, ^e^ Pi-Sigma, ^f^ Pi-Pi T-shaped, ^g^ Pi-Alkyl, ^h^ Alkyl, ^i^ Pi-Anion, ^j^ Pi-Cation, ^k^ Amide-Pi Stacked, ^l^ Pi-Pi Stacked.

**Table 7:** IC_50_ values (mM) obtained in ABTS and DPPH radical scavenging assays for DHCA, myrtenyl dihydrocaffeate, and BHT. Different letters within a column indicate statistically significant differences between samples (*p* < 0.05).

Compound	IC_50_ (mM)	
	ABTS	DPPH
DHCA	1.25 ± 0.03 A	0.11 ± 0.01 A
Myrtenyl dihydrocaffeate	1.19 ± 0.11 A	0.16 ± 0.02 B
BHT	1.14 ± 0.05 A	0.65 ± 0.02 C

**Table 8:** Maximum oxidation times (min) of rapeseed oil at 110, 120, 130, and 140 °C with and without the addition of DHCA, myrtenol, and myrtenyl dihydrocaffeate. Protection factors are given in parentheses.

Temperature (°C)	Control	DHCA	Myrtenol	Myrtenyl Dihydrocaffeate
140	18.73 ± 0.49 B	25.02 ± 0.56 (1.34) A	17.63 ± 0.12 (0.94) C	24.76 ± 0.54 (1.32) A
130	36.74 ± 1.84 B	49.94 ± 2.79 (1.36) A	38.76 ± 2.16 (1.05) B	50.33 ± 2.35 (1.37) A
120	79.05 ± 3.62 C	119.61 ± 1.27 (1.51) A	83.48 ± 0.52 (1.06) B	116.22 ± 1.81 (1.47) A
110	176.99 ± 1.15 B	246.46 ± 4.63 (1.39) A	178.81 ± 1.68 (1.01) B	243.73 ± 3.15 (1.38) A

Values are mean ± SD (*n* = 3). Different uppercase letters within the same row indicate significant differences among samples at the same temperature (*p* < 0.05). Statistical analysis was performed using one-way ANOVA followed by Tukey’s multiple comparisons test. Protection factor was calculated as the ratio of the oxidation time of oil containing the additive to that of the control sample at the same temperature.

**Table 9:** Kinetic parameters for the oxidation of rapeseed oil with and without the addition of DHCA, myrtenol, and myrtenyl dihydrocaffeate.

Sample	a	b	E_a_ (kJ/mol)	Z (min^−1^)	R^2^
Control	5162.31	−11.2302	98.84	1.70 × 10^11^	0.9990
DHCA	5318.19	−11.4763	101.83	2.99 × 10^11^	0.9975
Myrtenol	5303.61	−11.5791	101.55	3.79 × 10^11^	0.9987
Myrtenyl dihydrocaffeate	5291.11	−11.4129	101.31	2.59 × 10^11^	0.9986

**Table 10:** Growth inhibition zones (mm) determined by the disk diffusion assay on Mueller–Hinton agar for DHCA, myrtenol, and myrtenyl dihydrocaffeate.

Microorganism	Growth Inhibition Zone (mm)		
	DHCA	Myrtenol	Myrtenyl Dihydrocaffeate
*B. cereus* PCM 482	6.0 ± 0.0 Bb *	6.0 ± 0.0 Ab	12.8 ± 0.5 ABa
*B. subtilis* PCM 486	6.0 ± 0.0 Bb	6.0 ± 0.0 Ab	11.8 ± 1.0 BCa
*E. faecalis* PCM 2909	6.0 ± 0.0 Bb	6.0 ± 0.0 Ab	11.0 ± 0.8 CDa
*L. monocytogenes* PCM 2191	6.0 ± 0.0 Bb	6.0 ± 0.0 Ab	10.0 ± 0.8 Da
*S. aureus* PCM 2054	12.3 ± 1.0 Aa	6.0 ± 0.0 Ab	13.5 ± 0.6 Aa
*E. cloacae* PCM 2848	6.0 ± 0.0 Ba	6.0 ± 0.0 Aa	6.0 ± 0.0 Ea
*E. coli* PCM 2057	6.0 ± 0.0 Ba	6.0 ± 0.0 Aa	6.0 ± 0.0 Ea
*K. pneumoniae* PCM 1	6.0 ± 0.0 Ba	6.0 ± 0.0 Aa	6.0 ± 0.0 Ea
*S. marcescens* PCM 549	6.0 ± 0.0 Ba	6.0 ± 0.0 Aa	6.0 ± 0.0 Ea

* Values are presented as mean ± SD, where 6.0 mm indicates no detectable inhibition beyond the disk diameter. Different lowercase letters (a,b) indicate statistically significant differences within a row (between compounds for a given strain), whereas different uppercase letters (A–E) indicate statistically significant differences within a column (between strains for a given compound) (*p* < 0.05).

## Data Availability

The original contributions presented in this study are included in the article and Supplementary Materials. Further inquiries can be directed to the corresponding author (B.Z.).

## Supplementary Materials

The following supporting information can be downloaded at: https://www.mdpi.com/article/10.3390/biom16071034/s1, Figure S1. Predicted versus observed reaction yield (%) for the central composite design; Table S1. Analysis of variance (ANOVA) for the full quadratic response-surface model fitted to the CCD data for yield (%); Table S2. In silico predicted pharmacokinetic (ADMET) and toxicity properties of the synthesized ester and the parent compounds; Table S3. Biological activity spectrum of myrtenyl dihydrocaffeate; Table S4. Predicted targets of myrtenyl dihydrocaffeate; Table S5. Fatty acid composition of rapeseed oil (percentage of individual fatty acids in the total fatty acid content). SFA—saturated fatty acids; MUFA—monounsaturated fatty acids; PUFA—polyunsaturated fatty acids; Table S6. Reaction rate coefficients (k, min^−1^) of rapeseed oil oxidation at different temperatures; Table S7. Apparent inhibition of heat-induced protein denaturation by the tested compounds.

## Abbreviations

The following abbreviations are used in this manuscript:

ABTS	2,2′-azino-bis(3-ethylbenzothiazoline-6-sulfonic acid)
ADMET	absorption, distribution, metabolism, excretion, and toxicity
ALOX12	arachidonate 12-lipoxygenase/human 12-lipoxygenase
ANOVA	Analysis of Variance
ASE	affinity scoring estimation
BHT	butylated hydroxytoluene
BSA	bovine serum albumin
CALB	*Candida antarctica* lipase B
CCD	central composite design
CFU	colony-forming units
DHCA	Dihydrocaffeic acid
DMSO	dimethyl sulfoxide
DPPH	2,2-diphenyl-1-picrylhydrazyl
Ea	Activation energy
FAME	Fatty acid methyl ester
FID	flame ionization detector
GC-FID	gas chromatography with flame ionization detection
GBVI/WSA dG	generalized Born volume integral/weighted surface area binding free energy score
HSD	honestly significant difference
IC_50_	half-maximal inhibitory concentration
MBC	minimum bactericidal concentration
MIC	minimum inhibitory concentration
MOE	Molecular Operating Environment
MTBE	methyl *tert*-butyl ether
MUFA	monounsaturated fatty acids
NMR	nuclear magnetic resonance
PASS	Prediction of Activity Spectra for Substances
PBS	phosphate-buffered saline
PCM	Polish Collection of Microorganisms
PDB	Protein Data Bank
PDSC	Pressure differential scanning calorimetry
PF	Protection factor
P-gp	P-glycoprotein
PUFA	polyunsaturated fatty acids
ROS	Reactive oxygen species
RSD	relative standard deviation
RSM	response surface methodology
SFA	saturated fatty acids
SEA	Similarity Ensemble Approach
SILE	size-independent ligand efficiency
TLC	thin-layer chromatography
TRPV1	transient receptor potential cation channel subfamily V member 1
TMS	tetramethylsilane
VDss	steady-state volume of distribution
