# Supplementary material for: Integrated Experimental and Preliminary In Silico Study of Myrtenyl Dihydrocaffeate: Biocatalytic Synthesis Optimization, Antioxidant Evaluation, and Oxidative Stabilization of Rapeseed Oil
Source: Biomolecules. 2026 Jul 15;16(7):1034. doi: 10.3390/biom16071034 (PMC13407329; doi:10.3390/biom16071034)
Supplement: Supplementary file 1 [file biomolecules-16-01034-s001.zip › Supplementary Materials.pdf]

biomolecules

MDPI

Article

Integrated Experimental and Preliminary In Silico Study of Myrtenyl Dihydrocaffeate: Biocatalytic Synthesis Optimization, Antioxidant Evaluation, and Oxidative Stabilization of Rapeseed Oil

Bartłomiej Zieniuk [1,*], Jakub Gielmuda [2] and Chimaobi James Ononamadu [3]

[1] Department of Chemistry, Institute of Food Sciences, Warsaw University of Life Sciences-SGGW, 159C Nowoursynowska Str., 02-776 Warsaw, Poland
[2] Faculty of Biology and Biotechnology, Warsaw University of Life Sciences-SGGW, 159C Nowoursynowska Str., 02-776 Warsaw, Poland
[3] Department of Biochemistry and Forensic Science, Nigeria Police Academy Wudil, Kano P.O. Box 14830, Nigeria; ononamaducj@polac.edu.ng
* Correspondence: bartlomiej_zieniuk@sggw.edu.pl

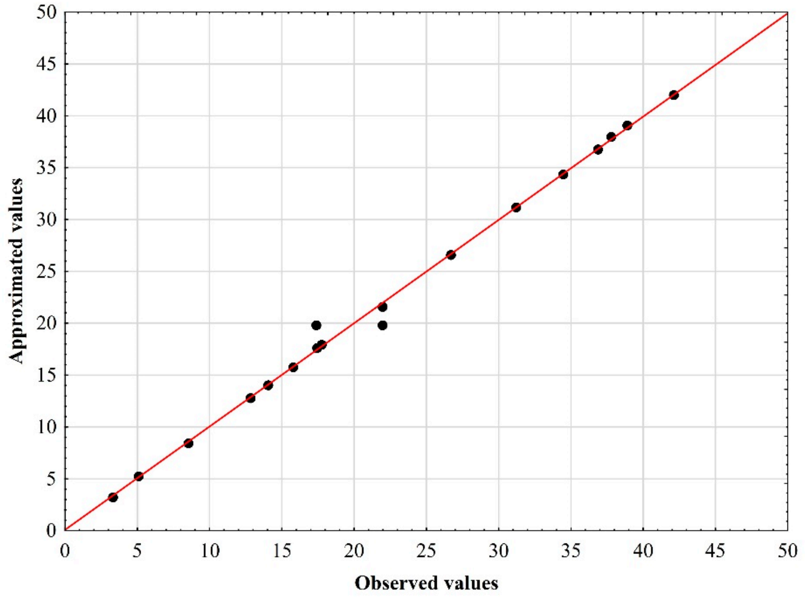

Figure S1. Predicted versus observed reaction yield (%) for the central composite design.

Table S1. Analysis of variance (ANOVA) for the full quadratic response-surface model fitted to the CCD data for yield (%).

**Source**	**SS**	**Df**	**MS**	***F-value***	***p-value***
(1)Temperature (°C)(L)	51.7882	1	51.7882	14.2258	0.0326
Temperature (°C)(Q)	207.0626	1	207.0626	56.8784	0.0048
(2)Enzyme concentration (%)(L)	485.1011	1	485.1011	133.2534	0.0014
Enzyme concentration (%)(Q)	1.5292	1	1.5292	0.4200	0.5631
(3)Time (h)(L)	322.4498	1	322.4498	88.5744	0.0025
Time (h)(Q)	59.9377	1	59.9377	16.4644	0.0270
(4)Myrtenol:DHCA Ratio(L)	563.8304	1	563.8304	154.8797	0.0011
Myrtenol:DHCA Ratio(Q)	43.0533	1	43.0533	11.8264	0.0413
1L×2L	233.0084	1	233.0084	64.0055	0.0041
1L×3L	77.2425	1	77.2425	21.2179	0.0192
1L×4L	155.2363	1	155.2363	42.6422	0.0073
2L×3L	54.6410	1	54.6410	15.0094	0.0304
2L×4L	59.6224	1	59.6224	16.3778	0.0272
3L×4L	30.5346	1	30.5346	8.3876	0.0627
Error	10.9213	3	3.6404		
Total SS	2481.2492	17			

*Explanations: Factors: 1—incubation 2—enzyme concentration (%), 3—time (h), 4—myrtenol:DHCA ratio. L—linear (first-order) term; Q—quadratic (squared) term; and 1L×2L denotes the two-factor interaction between the linear effects of factors 1 and 2 (analogously for other pairs). Abbreviations: SS – Sum of Squares; df - degrees of freedom; MS – Mean Square.

Table S2. *In silico* predicted pharmacokinetic (ADMET) and toxicity properties of the synthesized ester and the parent compounds.

Category	Parameter	Unit	Myrtenol	DHCA	Myrtenyl dihydro-caffeate
Absorption	Water solubility	log mol/L	-2.369	-1.764	-3.491
	Caco-2 permeability	log Papp (10^{-6} cm/s)	1.476	0.000	1.078
	Intestinal absorption (human)	%	94.799	55.476	93.885
	Skin permeability	log Kp	-2.366	-2.734	-3.122
	P-glycoprotein substrate	Yes/No	No	Yes	Yes
	P-glycoprotein I inhibitor	Yes/No	No	No	No
	P-glycoprotein II inhibitor	Yes/No	No	No	No
	Bioavailability score	–	0.55	0.55	0.55
Distribution	VDss (human)	log L/kg	0.489	-0.372	0.315
	Fraction unbound (Fu)	–	0.496	0.364	0.103
	BBB permeability	log BB	0.769	-0.869	-0.366
	CNS permeability	log PS	-2.521	-3.347	-2.365
Metabolism	CYP2D6 substrate	Yes/No	No	No	No
	CYP3A4 substrate	Yes/No	No	No	No
	CYP1A2 inhibitor	Yes/No	No	No	No
	CYP2C19 inhibitor	Yes/No	No	No	No
	CYP2C9 inhibitor	Yes/No	No	No	No
	CYP2D6 inhibitor	Yes/No	No	No	No
	CYP3A4 inhibitor	Yes/No	No	No	No
Excretion	Total clearance	log mL/min/kg	0.054	0.319	0.128
	Renal OCT2 substrate	Yes/No	No	No	No
Toxicity	AMES toxicity	Yes/No	No	No	No
	Max. tolerated dose (human)	log mg/kg/day	0.583	0.336	-0.250
	hERG I inhibitor	Yes/No	No	No	No
	hERG II inhibitor	Yes/No	No	No	Yes
	Oral rat acute toxicity (LD_{50})	mol/kg	1.805	1.995	2.328
	Oral rat chronic toxicity (LOAEL)	log mg/kg/day	1.816	2.049	1.767
	Hepatotoxicity	Yes/No	No	No	No
	Skin sensitisation	Yes/No	Yes	No	No
	T. pyriformis toxicity	log µg/L	0.263	0.028	0.635
	Minnow toxicity	log mM	1.639	1.536	0.354

Table S5. Fatty acid composition of rapeseed oil (percentage of individual fatty acids in the total fatty acid content). SFA - saturated fatty acids; MUFA - monounsaturated fatty acids; PUFA - polyunsaturated fatty acids.

Fatty acid	Common name	Content (%)
C16:0	Palmitic acid	4.58 ± 0.21
C18:0	Stearic acid	2.20 ± 0.09
C18:1	Oleic acid	62.09 ± 0.45
C18:2	Linoleic acid	19.84 ± 0.06
C18:3	Linolenic acid	8.95 ± 0.25
C20:0	Arachidic acid	0.68 ± 0.01
C20:1	Eicosenoic acid	1.68 ± 0.03
SFA		7.46 ± 0.29
MUFA		63.77 ± 0.48
PUFA		28.78 ± 0.18

Table S6. Reaction rate coefficients (k, min^{-1}) of rapeseed oil oxidation at different temperatures.

Sample	110 °C	120 °C	130 °C	140 °C
Control	0.005681	0.012509	0.026484	0.054072
DHCA	0.003923	0.008847	0.019159	0.039969
Myrtenol	0.005426	0.012208	0.026382	0.054927
Myrtenyl dihydrocaffeate	0.003989	0.008957	0.019322	0.040159

Table S7. Apparent inhibition of heat-induced protein denaturation by the tested compounds.

Compound	Inhibition of protein denaturation (%)			
	Concentration (mM)			
	**0.01**	**0.25**	**0.50**	**1.00**
DHCA	1.18 ± 0.41 Bc	6.51 ± 0.52 Ca	3.14 ± 0.58 Cb	1.79 ± 0.35 Bc
Myrtenol	0.47 ± 0.20 Bc	1.99 ± 0.49 Bb	5.57 ± 0.51 Ba	0.45 ± 0.27 Cc
Myrtenyl dihydrocaffeate	2.39 ± 0.33 Ad	8.27 ± 0.42 Ac	14.94 ± 0.45 Aa	10.39 ± 0.38 Ab

Values are mean ± SD (n = 3). Different lowercase letters within the same row indicate significant differences among concentrations for the same compound ($p < 0.05$). Different uppercase letters within the same column indicate significant differences among compounds at the same concentration ($p < 0.05$). Statistical analysis was performed using one-way ANOVA followed by Tukey's multiple comparisons test.
